# Transient Evolution of the Built-in Field at Junctions of GaAs

**DOI:** 10.1021/acsami.0c11474

**Published:** 2020-08-18

**Authors:** Xihan Chen, Ryan T. Pekarek, Jing Gu, Andriy Zakutayev, Katherine E. Hurst, Nathan R. Neale, Ye Yang, Matthew C. Beard

**Affiliations:** † Materials and Chemical Science and Technology Directorate, National Renewable Energy Laboratory, Golden, Colorado 80401, United States; ‡ Department of Chemistry and Biochemistry, San Diego State University, San Diego, California 92182, United States; § State Key Laboratory of Physical Chemistry of Solid Surfaces, College of Chemistry and Chemical Engineering, Xiamen University, Xiamen, Fujian 361005, P. R. China

**Keywords:** ultrafast spectroscopy, solar energy conversion, photoelectrochemical cell, semiconductor photoelectrode, carrier dynamics

## Abstract

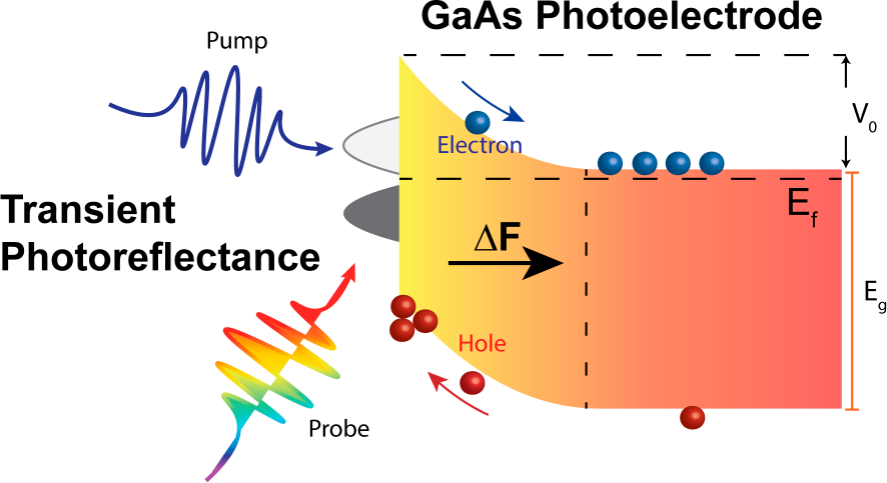

Built-in electric fields at semiconductor junctions are vital for optoelectronic and photocatalytic applications since they govern the movement of photogenerated charge carriers near critical surfaces and interfaces. Here, we exploit transient photoreflectance (TPR) spectroscopy to probe the dynamical evolution of the built-in field for n-GaAs photoelectrodes upon photoexcitation. The transient fields are modeled in order to quantitatively describe the surface carrier dynamics that influence those fields. The photoinduced surface field at different types of junctions between n-GaAs and n-TiO_2_, Pt, electrolyte and p-NiO are examined, and the results reveal that surface Fermi-level pinning, ubiquitous for many GaAs surfaces, can have beneficial consequences that impact photoelectrochemical applications. That is, Fermi-level pinning results in the primary surface carrier dynamics being invariant to the contacting layer and promotes beneficial carrier separation. For example, when p-NiO is deposited there is no Fermi-level equilibration that modifies the surface field, but photogenerated holes are promoted to the n-GaAs/p-NiO interface and can transfer into defect midgap states within the p-NiO resulting in an elongated charge separation time and those transferred holes can participate in chemical reactions. In contrast, when the Fermi-level is unpinned via molecular surface functionalization on p-GaAs, the carriers undergo surface recombination faster due to a smaller built-in field, thus potentially degrading their photochemical performance.

## Introduction

Semiconductor electrodes are widely used in photoelectrochemical (PEC) cells that are capable of directly converting sunlight into chemical fuels.^1−10^ At the electrode surfaces and/or interfaces and junctions, a built-in electric field can form as a result of Fermi-level equilibration. The built-in field plays a vital role in determining the PEC performance as it affects the photocurrents in the devices and the resulting chemical reactions occurring at the electrode or catalyst surfaces,^11,12^ as well as controlling surface-carrier recombination. To increase stability and tune the energetics^13,14^ of widely used photoelectrodes (e.g., Si, GaAs, GaP, and GaInP_2_), inorganic surface layers such as TiO_2_ are typically deposited onto the photoelectrode surfaces.^15^ In particular, gallium arsenide (GaAs), which is one of the most widely used semiconductors for high-efficiency solar and PEC cells, has been studied in contact with TiO_2_,^16^ NiO,^17^ graphene,^18^ and SrTiO_3_.^19^ For example, with amorphous TiO_2_ coating on GaAs, 15 mA/cm^2^ photocurrent at 1V bias against RHE at pH 14.^15^ Similarly, with SrTiO_3_ coating on GaAs, a photocurrent of ∼6 mA/cm^2^ can be achieved at 0 V vs RHE under 1 sun illumination. An IPCE of >40% can be observed for light wavelengths in the range of 500 to 800 nm at 0 V vs RHE.^19^ Sometimes, Fermi-level equilibration between the electrode and the surface layer can significantly impact the original surface built-in field,^20^ which in-turn modifies the interfacial charge carrier dynamics at these junctions. Thus, understanding the transient evolution of such built-in electric fields upon optical photoexcitation can offer insights into how to design systems with high photon-to-chemical conversion efficiency in photoelectrode systems. Recently transient photoreflectance (TPR) spectroscopy has been developed and applied to directly measure the evolution of the built-in surface electric fields at promising photoelectrode junctions such as p-GaInP_2_,^13^ BiVO_4_,^21^ and Si.^22^ Using this method, the charge carrier dynamics that impact these fields can therefore be extracted with subpicosecond temporal resolution and without the need to attach wires (i.e., the technique is a noncontact probe of built-in electric fields).

Here, we employ TPR to investigate the photoinduced surface field dynamics at different GaAs surfaces and junctions with various overlayers. The dynamics of the surface field are modeled by considering both charge-carrier drift and diffusion, from which we extract the diffusion constant of the minority carriers at the edge of the depletion zone. The photoinduced dynamics of the surface field for several different n-GaAs junctions exhibit identical evolution patterns, indicative of a surface Fermi-level pinning that locks the initial charge-carrier dynamics irrespective of the identity of the interfacial material. However, these carrier dynamics can be modified by a molecular surface modification that unpins the Fermi-level. We find that surface functionalization with 4-(trifluoromethyl)phenyl groups on p-GaAs results in an unpinned Fermi-level and a resulting lower built-in field, decreasing photogenerated charge separation and resulting in faster surface recombination.

## Transient Photoreflectance on Bare n-GaAs

**Figure 1:**
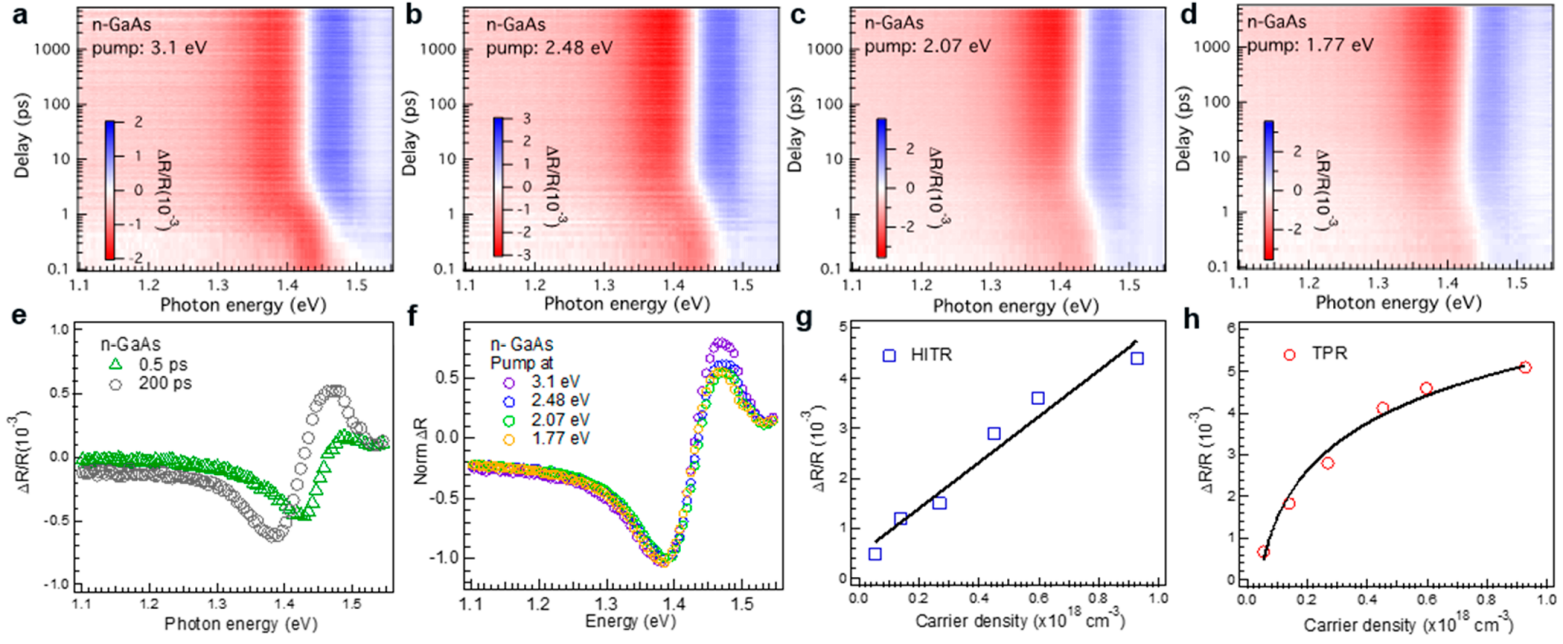
Transient Photoreflectance (TPR) spectra of n-GaAs in air. Pseudocolor image for n-GaAs pumped at photon energies of (a) 3.1 eV, (b) 2.48 eV, (c) 2.07 eV, and (d) 1.77 eV. Intensities of red and blue in pseudocolor images represent the magnitude of the reduced and increased reflectance, respectively. (e) Snapshots of TPR spectra at delays of 0.5 ps (green) and 200 ps (black) pumped at 2.48 eV with photocarrier density around 1 × 10^17^cm^–3^. The spectrum at early time delay shows a negative peak centered at 1.43 eV and evolves into antisymmetric peaks at later time delay. (f) Normalized transient reflection spectra of n-GaAs pumped at 3.1 eV (purple), 2.48 eV (blue), 2.07 eV (green), and 1.77 eV (orange) at 200 ps delay show that the spectra are invariant with different pump excitation energy. Amplitude of photogenerated signal recorded at (g) 0.5 ps and (h) 200 ps as a function of charge carrier density. Linear relationship observed between signal recorded at 0.5 ps delay time and carrier density. Logarithm dependence is observed for the signal recorded at 200 ps and carrier density. The early part of the photogenerated signal is then assigned to hot carriers (HITR), and the late part of the signal is assigned to the reflectance change induced by field modulation (TPR).

Figure 1a–d shows pseudocolor images of TPR spectra of bare n-GaAs under various pump photon energies. The horizontal and vertical axes indicate the probe photon energy and the delay time, respectively. The color intensity represents the spectral magnitude (see scale bar). At early pump–probe delays (i.e., 0.1–1 ps), an asymmetric spectral feature with a large negative and a smaller positive part is observed (Figure 1e, green triangles), and the magnitude of the negative part increases for larger pump photon energies, suggesting that this asymmetric feature can be associated with the presence of hot-carriers. At longer pump–probe delays, the asymmetric feature evolves into a pair of antisymmetric peaks that straddle the bandgap energy (Figure 1e, black circles), whose shape is independent of the pump photon energy (Figure 1f). During the spectral evolution there is a gradual increase in the magnitude of the antisymmetric peaks with concurrent decrease of the asymmetric feature.

In semiconductors, photoinduced reflectance measures the change in the complex frequency-dependent refractive index Δ*ñ*(ω), which at the bandedge is dominated by the real part of *ñ*. There are many effects that could be responsible for the photoinduced changes in *n*, including Pauli-blocking,^23−26^ spectral broadening due to carrier–carrier interactions,^22,27^ a spectral shift due to bandgap renormalization,^28−30^ photon-induced thermal effects,^31,32^ a Drude response resulting from the presence of free-carriers,^22,33^ and the Franz–Keldysh effect which arises from the photomodulation of a built-in surface electric field.^13,34^ We can distinguish between these effects by their dependence on pump-fluence. Only bandgap renormalization and the Franz–Keldysh effect give rise to a nonlinear relationship between the transient spectral signal and the carrier density in the low excitation regime (<10^18^ cm^–3^).^22,23,28,32^ The transient signal arising from bandgap renormalization should be proportional to the cube root of the carrier density,^28,29^ while the signal due to the Franz-Keldysh effect is proportional to the logarithm of the carrier density.^13^

Thus, to determine the origin of the transient spectra at early and late pump–probe delay times, we performed intensity dependent measurements pumping at 2.48 eV. The average carrier densities were determined from the pump fluence and the absorption coefficient at 2.48 eV (see Supporting Information, SI, for calculations). The amplitude of the asymmetric feature at early pump–probe delay times (∼0.5 ps) is linearly proportional to the carrier density (Figure 1g) and thus we assign this feature to the presence of hot carriers (HITR, see discussion below). Alternatively, the amplitude of the antisymmetric peak at long delay (∼200 ps) has a logarithmic dependence on the input carrier density (Figure 1h) and thus, we assign it to a modulation of the surface electric field (TPR, see discussion below).

Considering that the hot-carrier associated spectrum (HITR) prevails for only a short duration (<2 ps), thermal effects can be excluded because the lattice temperature cannot respond prior to the relaxation of hot-carriers. The spectral shape of the HITR is distinct from that due to a Drude response, which should exhibit a broad negative signal extending into the deep sub-bandgap region with a gradually increasing amplitude.^22,33^ Since this is different from the observed spectral shape (Figure 1e), the Drude response cannot account for the HITR. Pauli-blocking due to carrier occupation at the band edges should not exhibit a dependence on the pump photon energy, and Pauli blocking of hot-carriers should only weaken the optical transitions above the bandgap and not at the bandedge. Thus, Pauli-blocking cannot be responsible for HITR. Therefore, we attribute the HITR spectrum to hot-carrier induced spectral shifting or broadening of the bandedge.

On the basis of the relationship between the signal magnitude and input carrier density, the antisymmetric feature that occurs at longer pump–probe delay times is attributed to the Franz–Keldysh effect. Upon optical excitation, photo generated electrons and holes that reside within the surface depletion region quickly drift toward the bulk and surface, respectively, driven by a built-in surface field because of Fermi-level equilibration in the dark.^35^ The separation of the photocarriers will modulate that built-in field, resulting in a TPR signal according to the Franz–Keldysh theory.^13^ Therefore, in the following we focus our discussion on the TPR signal occurring at pump–probe delay times >1 ps.

## Simulating Transient Surface Field Dynamics with Carrier Dynamics

**Figure 2:**
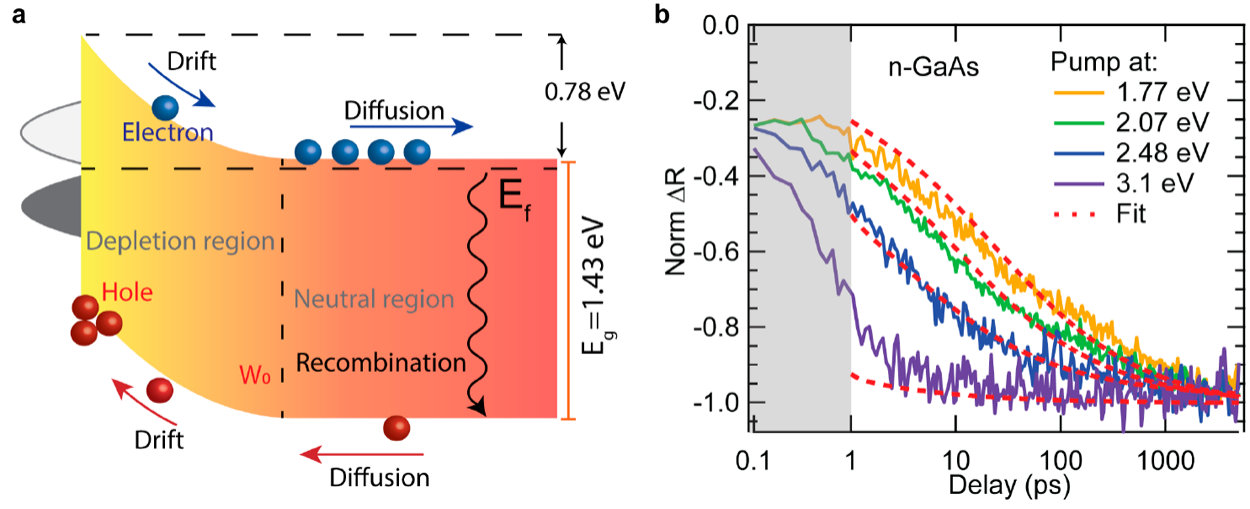
(a) Schematic illustration of band diagram at n-GaAs surfaces in air. Charge carriers will drift, diffuse, and recombine after photoexcitation. The band bending can be determined from modeling the dynamics of the surface field. (b) Dynamics of the transient modulation field due to the charge separation at the depletion region in air. These dynamics are represented by the kinetic traces of the antisymmetric negative peak. The corresponding pump photon energies are also indicated. The red dashed traces represent the fits from diffusion model. Note that our model does not cover the measured kinetics for *t* < 1 ps. This is due to the hot carrier or drift effect (*t* < 1 ps) that are not accounted for in our model.

At semiconductor surfaces, carriers can drift, diffuse, and recombine (Figure 2a). Drift and diffusion cause a build-up (a rise in the observed signal from 0 to −1 in Figure 2b) of the modulated electric field, while carrier recombination results in a decrease (a decay of the observed signal from −1 to 0) in the photogenerated field. Drift processes generally complete on a time scale of a few ps depending on the field strength, depletion width, and carrier mobilities.^36^ For simplicity, we assume here that the dynamic behavior of carriers located within the depletion region is dominated by drift, while the dynamic behavior of carriers located beyond the depletion region (neutral region) is dominated by diffusion. Carriers residing in the neutral region can diffuse either into the depletion region or they can diffuse toward the bulk, driven by a concentration gradient.
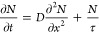
1where *N* is the carrier concentration, *D* is the diffusion constant for minority carriers, i.e., holes, τ is the bulk carrier lifetime.

In the case of an n-type semiconductor, such as n-GaAs studied here, due to an upward band bending near the surface, photogenerated holes diffuse into the depletion region where they are swept to the surface by the built-in field, while photogenerated electrons are swept into the bulk and then diffuse further into the bulk. The resulting transient electric field strength then depends upon the number of photogenerated holes that diffuse from the bulk into the depletion region. The opposite would be true for a p-type semiconductor. Thus, the resulting dynamics inform upon the number of minority photogenerated carriers that enter the depletion region. A common approach in modeling similar carrier dynamics is to assume a virtual boundary surface that divides the depletion from the neutral region, and the carrier flux at this boundary is characterized by a charge-carrier velocity, *S*_v_ (see eqs 2 and 3). Thus, we can define a boundary condition that the carrier flux (*J*) must meet at that virtual boundary surface,
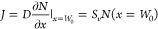
2
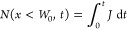
3where *W*_0_ is the depletion region width, and *S*_v_ is the thermal velocity of holes (minority carriers) at the virtual boundary of the depletion region. Equation 3 describes those carriers that cross the depletion region and are swept to the semiconductor surface and thus add to the transient field. The resulting TPR signal is proportional to the logarithm of the carrier concentration (Figure 1h) that reach the surface (eq 3).
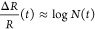
4where Δ*R* and *R* are measured signals as shown in Figures 1 and 2.

As a result of the different absorption coefficient at the different pump wavelengths, we generate different initial carrier distribution profiles near the surface for each pump-excitation wavelength, which is defined by the following:
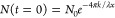
5where *k* is the imaginary part of the refractive index, and λ is the pump wavelength (see SI for complex refractive index). The penetration depth, δ, is then defined to be δ = λ/4*πk*. In the bare n-GaAs experiment (Figure 2b) the pump photon energies are set to be the following values, 3.1, 2.48, 2.08, and 1.77 eV, corresponding to a carrier generation depth below surfaces of 13, 50, 108, and 185 nm. For the smallest photon-carrier generation depth of 13 nm, the photocarriers are all generated within the depletion region and thus all feel the presence of the surface field. Therefore, the generated carriers are quickly separated by the field which correspond to the fast rise of the measured signal in Figure 2b under 3.1 eV pump photons. For the largest carrier generation depth of 185 nm, some of the carriers are generated in the neutral region and thus diffuse based on their concentration gradient, while those generated in the depletion region undergo drift that sweep holes to the surface and electrons into the bulk. The diffusion process is much slower compared to the drift process which results in a slower rise in the dynamics of Figure 2b under 1.77 eV pump photons.

These five equations can describe the measured TPR dynamics. Since in this case the depletion width and the light penetration depth are much smaller than the wafer thickness we can solve for *J*, the flux of carriers passing from the neutral region into the depletion region analytically (see section in the SI for the analytic expression). The initial conditions are defined by the initial carrier distribution profiles based on eq 5, and simulates the modulation field dynamics, which is represented by the spectral kinetics of the TPR feature (near the negative part of the antisymmetric peak). In addition to charge separation within the depletion region, holes that diffuse toward the virtual boundary (eq 3) also contribute to the buildup of the modulation field and thus the observed signal and accounts for the growth of the kinetic traces. In the modeling, several input parameters are derived from literature value such as *S*_v_ (1.8 × 10^7^ cm·s^–1^, calculated from ref (37)) and *D* (7.5 cm^2^*S*^–1^ derived from ref (38)). The bulk carrier lifetime τ is generally on the order of hundreds of ns to a few microseconds^39^ (∼3 μs) and therefore will not heavily affect the kinetics within the 5 ns probe window used here. In our simulation, the unknown parameter is *W*_0_ (the depletion width), which is allowed to vary using a global nonlinear least-squares method to find the best-fit value that best describes the measured kinetics (Figure 2b). From our simulation, we obtained *W*_0_ of 35 nm ±5 nm which corresponds to a band bending (*V*_0_) of ∼0.78 V ± 0.15 V that is consistent with values reported in literature from other types of measurements.^40,41^ The depletion region width can be expressed as follows:
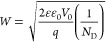
6where ϵ is the relative permittivity of GaAs (12.95),^42^ ϵ_0_ is the vacuum permittivity (8.85 × 10^–12^ F m^–1^), *q* is the elementary charge (1.6 × 10^–19^*C*), and *N*_D_ is the doping density (9.1 × 10^17^ cm^–3^). The maximum intrinsic field strength (*F*) can also be extracted (*F* = *V*_0_/*ϵW*) and we find ∼16 ± 3 kV cm^–1^.

## Dynamics of Surface Field Across Different Junctions

**Figure 3:**
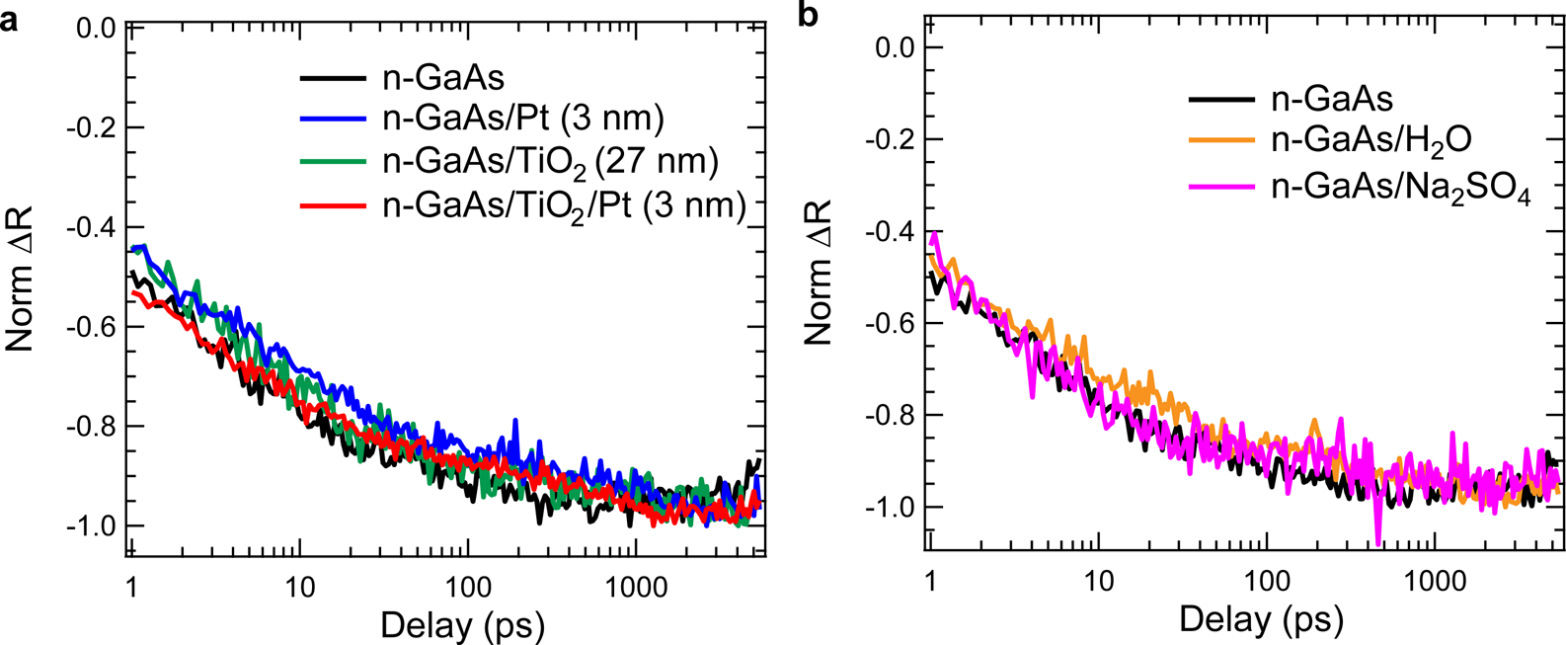
Transient kinetics of the surface field for bare n-GaAs and (a) n-GaAs/Pt in air, n-GaAs/TiO_2_ in air, n-GaAs/TiO_2_/Pt in air and (b) bare n-GaAs, n-GaAs/H_2_O at open circuit, n-GaAs/0.1 M Na_2_SO_4_ at open circuit with 2.48 eV pump. The incident photocarrier density is kept the same around 1.35 × 10^17^ cm^–3^.

We investigated several widely used n-GaAs photoelectrode junctions. For these experiments we held the pump energy at 2.48 eV. We first investigate several different types of common n-GaAs junctions, such as n-GaAs/TiO_2_, n-GaAs/Pt (Schottky-junction), and n-GaAs/electrolyte (Schottky-junction). The kinetics of the primary transient electric fields for delay times <5000 ps are plotted in Figure 3a,b. Interestingly, the primary kinetics are invariant to the interfacial TiO_2_, Pt coating (Figure 3a) or when in contact with an electrolyte, (Figure 3b) indicating the same charge separation dynamics occur for these junctions as for the bare n-GaAs. The invariance of the transient electric field dynamics across different n-GaAs/junctions can be associated with the presence of Fermi-level pining. In the presence of a high-density of surface states due to the formation of native surface oxides, the Fermi-level can be pinned at the gap between occupied and empty surface states,^40,43^ and as a result of equilibration with carriers residing in the bulk the resulting built-in electric field is independent of the contacting material. With a fixed surface field, the primary charge separation process is invariant to the contacting material since it does not significantly modify the intrinsic surface states. With the relatively large band bending (0.78 V) for bare n-GaAs even before additional layers are deposited, the charge separation will also be efficient when a catalyst (Pt) or inorganic layer (TiO_2_) is coating the underlying n-GaAs.

**Figure 4:**
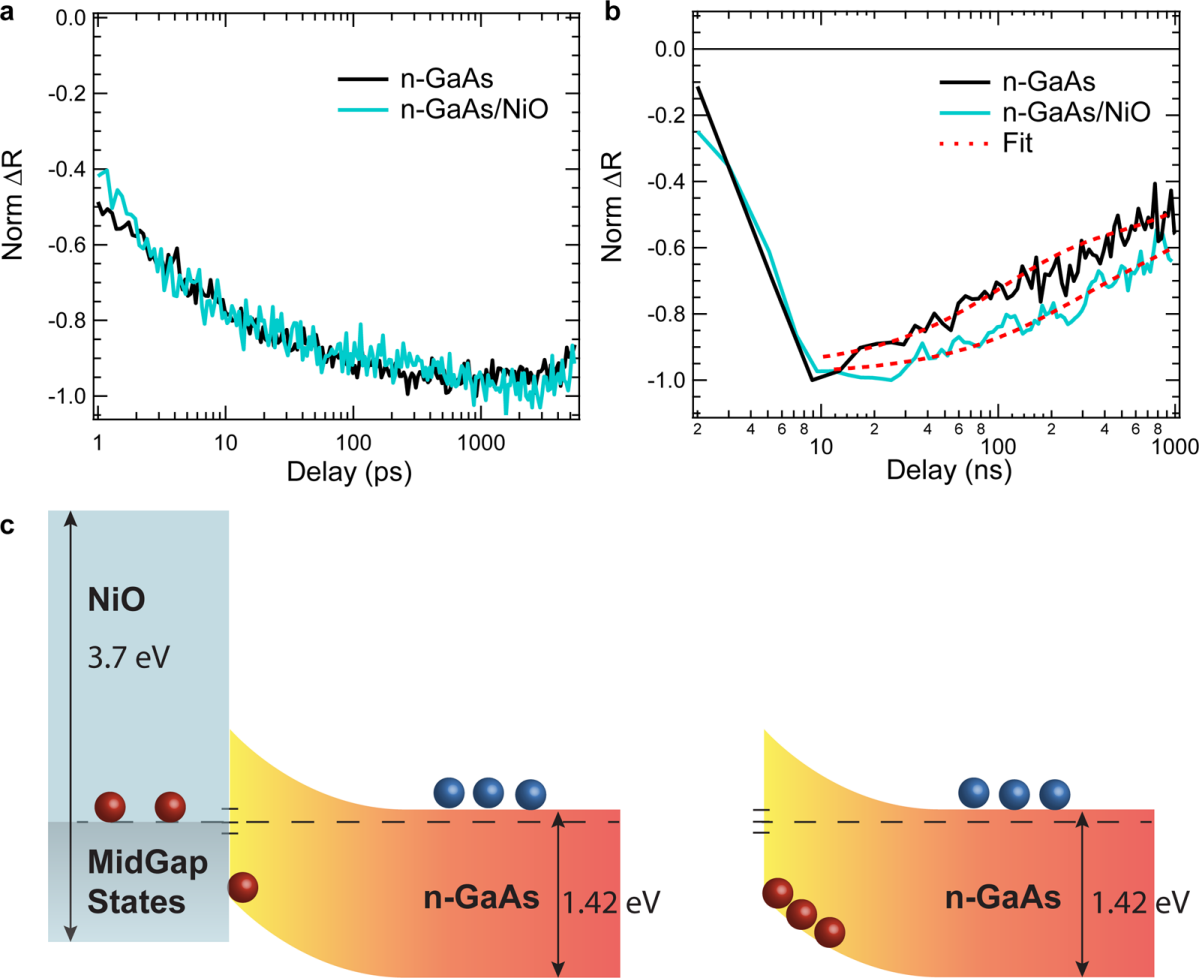
Transient kinetics of surface field probed at 1.4 eV for bare n-GaAs and n-GaAs/NiO in air at (a) fs to 5 ns (b) 5 to 1000 ns time scale with 2.48 eV pump. At ultrafast time scale up to 5 ns, the kinetics are invariant with or without NiO. After 5 ns, n-GaAs with NiO decays slower compared with bare n-GaAs. The kinetics can be fitted with a double exponential decay (red dashed line). N-GaAs shows an average lifetime() of ∼3 μs and n-GaAs/NiO shows a lifetime of ∼3.9 μs. (c) Band diagram for n-GaAs/NiO and bare n-GaAs. The relative band positions of NiO and GaAs are taken from published literature.^44^ With NiO coating, hole transfer occurs which create a charge separated state and increase carrier lifetime.

Apart from the above overlayers, we also investigated a n-GaAs/p-NiO junction. The TPR kinetics for n-GaAs/p-NiO junctions (probed at 1.4 eV) are plotted in Figure 4a. NiO is a well-known p-type wide bandgap semiconductor and has been widely used on semiconductors surfaces as a catalyst to enhance the resulting photoelectrochemical performance.^16,17^ A n-GaAs/p-NiO heterojunction could form p-n heterojunction, that would enhance the charge carrier extraction and enhance the photocurrent, as well as prevent harmful self-oxidation that could degrade the underlying GaAs. The rising kinetics after 1 ps represent the formation of a transient electric field due to charge separation as described and modeled in the previous section and is invariant with or without the p-NiO coating. This result suggests that the deposition of p-NiO does not alter the intrinsic surface electric field nor modify the depletion region width from that found for bare n-GaAs. This is in contrast to our previous study of p-GaInP_2_ surfaces that are overcoated with n-TiO_2_.^13^ In that case a p–n junction forms producing a very large surface/interfacial field (>100 kV cm^–1^) that quickly separates carriers.

However, at much longer pump–probe delay times (>20 ns), the kinetics for n-GaAs/p-NiO decay exhibit a slower recombination of the carriers than that observed for bare n-GaAs (Figure 4b). The observed decay on these longer time scales is a result of carrier recombination that then diminishes the transient field, restoring the original surface field. The kinetics can be fitted with double exponential function. The fitted lifetime for bare n-GaAs is ∼3 μs and for the n-GaAs/p-NiO sample we find ∼3.9 μs. We speculate that the slightly longer recombination time results from a hole transfer from the n-GaAs surface states to p-NiO midgap states since the valence band of NiO is a few hundred meV below the GaAs.^45^ The resulting charge separated state prolongs the carrier lifetime by reducing the recombination rate. Therefore, with a p-NiO coating, photogenerated charge in the n-GaAs can be extracted efficiently through those midgap states (Figure 4c). To summarize, the charge separation kinetics at early times is independent of p-NiO coating (Figure 4a), but the charge recombination is slower (Figure 4b).

## Unpinning the Fermi-Level with Molecular Surface Functionalization

**Figure 5:**
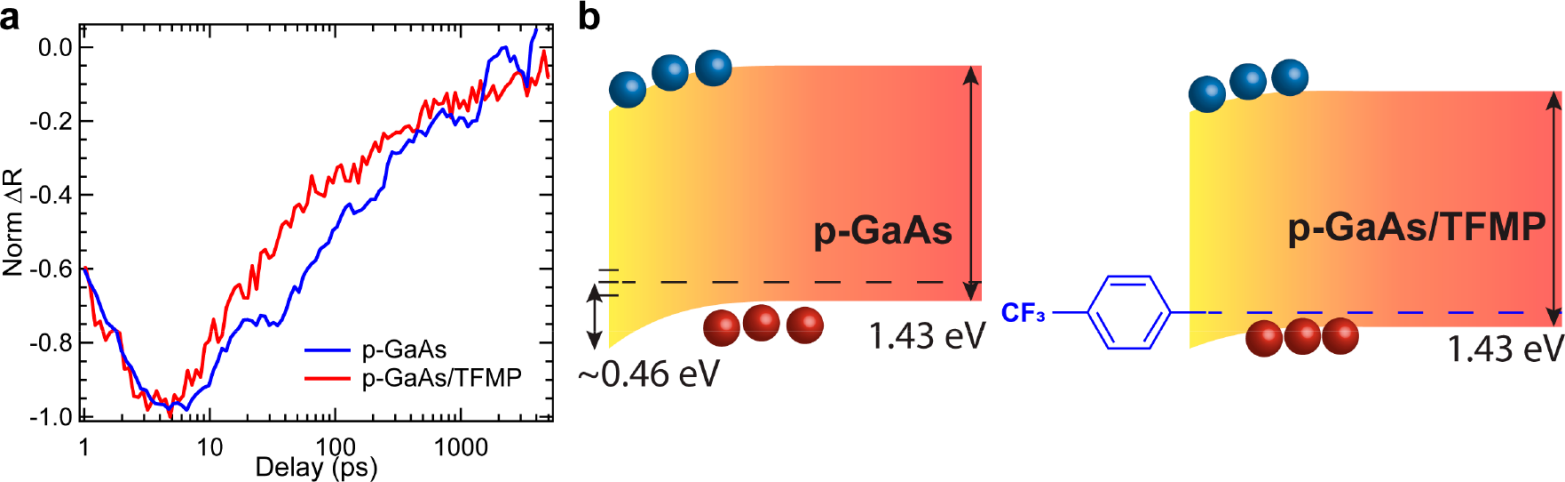
(a) Transient kinetics probed at 1.4 eV for bare p-GaAs (denoted p-GaAs) and 4-(trifluoromethyl) phenyl terminated p-GaAs (denoted p-GaAs/TFMP) under 2.48 eV pump in air. The carrier density is kept low around 3 × 10^17^*cm*^–3^. (b) Band diagram for p-GaAs and p-GaAs/TFMP. Compare with p-GaAs, the attachment of TFMP shifts the Fermi-level which results in a decrease in surface field and accelerated surface recombination.

TPR on n-GaAs indicates a pinned Fermi-level regardless of the deposition of the various surface layers studied here. We also observe this effect for p-type GaAs, with some differences. Unlike n-GaAs, the dynamics in the unfunctionalized, bare p-GaAs exhibits both a rise and decay feature (probed at 1.4 eV and pumped at 2.48 eV). (Figure 5a) As discussed above, the rising dynamics (going more negative) represents charge separation and the decay dynamics (back to zero) represents charge recombination. Thus, compared with the n-GaAs sample studied above, charge carriers in p-GaAs separate and recombine much faster, resulting in a decay of transient field within a ∼1 ns. This faster decay of the field could indicate that for similar doping densities, n- compared to p-GaAs surfaces, the field strength and band bending are smaller for p-GaAs than for n-GaAs. (The fast decay could also due to the larger surface recombination velocity in p-type due to a faster electron thermal velocity.) A similar phenomenon has been observed at GaAs/metal junctions where p-type GaAs has half the band bending compared to that found for n-GaAs^40^ (e.g., 0.95 V at n-GaAs/Au junction vs 0.48 V at p-GaAs/Au junction). For our n- and p-GaAs samples, the Fermi-level can be estimated as follows:
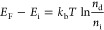
7where *E*_i_ is the intrinsic Fermi-level that lies in the middle of the bandgap, *k*_b_*T* is the thermal energy at room temperature (0.0259 eV), and *n*_d_ is the doping density near 10^18^ cm^–3^, and *n*_i_ is the intrinsic doping density (2.1 × 10^6^ cm^–3^). On the basis of eq 7, the Fermi-levels for n- and p-types GaAs used in our experiments are ∼0.026 eV below the conduction band and above the valence band. Therefore, with the 2:1 band bending ratio reported in the literature for n vs p type and 1.43 eV bandgap, the built-in voltage can be estimated to be 0.92 and 0.46 V for n- and p-type GaAs, respectively. This estimated value is a little higher than the measured band bending of 0.78 V for n-type shown in Figure 2 but still in good agreement. The smaller built-in voltage for the p-type gives rise to a smaller built-field strength, a smaller depletion region, less charge separation, and a resulting faster charge recombination time as observed. To verify we simulated the charge separation dynamics for the p-type GaAs with three different pump wavelengths using literature values for the electron thermal velocity and diffusion constant and a built-in field that is two times smaller and resulting depletion width of ∼25 nm and find good agreement with the measured data (see Figure S5).

To investigate the effect of Fermi-level unpinning to the surface field, we investigated a molecular surface functionalization of organic molecules that are known to alter the surface energetics via dipolar effects.^46−48^ For example, we previously^47^ demonstrated that the use of 4-(trifluoromethyl)phenyl (TFMP) on p-GaAs surfaces shifts the photocurrent onset potential to more positive values vs RHE by ∼89 mV at pH of 2, suggesting some degree of surface unpinning. Here we prepared identical samples and study the modulation to the field dynamics. Figure 5a shows the transient kinetics probed at 1.4 eV for bare p-GaAs and p-GaAs/TFMP. The bare p-GaAs surface exhibits a slower charge-carrier decay compared with the TFMP-terminated surface, indicating that there is a larger intrinsic surface field present for the bare p-GaAs surface. A larger built-in field is able to separate charges more efficiently and also suppresses surface carrier recombination, resulting in a slower decay of the transient electric field.^49^ From our measurements, the attachment of TFMP molecules shifts the surface Fermi-level at the cost of a decrease in surface built-in field, which could decrease the current density and efficiency of a photoelectrochemical system (Figure 5b). Such a phenomenon has been observed with a p-GaInP_2_ photoelectrode where surface functionalization beneficially shifts the onset potential but decreases the overall photocurrent density.^46^ We attribute the lower photocurrent density to an increase in surface carrier recombination resulting from a weaker built-in field caused by the molecular dipoles. In the future, a similar surface unpinning treatment could be applied to n-GaAs, and the TPR spectroscopy can used to determine the degree of surface Fermi-level unpinning.

## Conclusions

We have employed TPR to probe the interfacial electric field dynamics for various GaAs surfaces. The electric field dynamics can be modeled with carrier diffusion to the edge of depletion zone after which minority carriers are swept to the surface by the built-in field, such charge separation reduces the surface field. From the analysis the depletion region width can be extracted, and the build-in voltage can be calculated. We find for the n-GaAs samples studied there is an invariance of the interfacial built-in electric field across various n-GaAs junctions (n-TiO_2_, Pt, electrolyte and p-NiO) as a result of Fermi-level pinning. Interestingly, photoinduced hole transfer occurs between n-GaAs/p-NiO which results in elongated charge separation time. We find that upon unpinning of the surface of p-GaAs by a surface molecular functionalization there is a decrease in charge separation efficiency and shorter surface carrier lifetimes. These results demonstrate the use of TPR to elucidate the surface carrier dynamics in GaAs junctions.

## Supporting Information Available

Materials and experimental methods; refractive index of GaAs; XRD of NiO; carrier density calculation; carrier kinetics under two difference fluences; and TPR spectra of n- and p-GaAs (PDF)
