# Supplementary material for: Transient Evolution of the Built-in Field at Junctions of GaAs
Source: ACS Appl Mater Interfaces. 2020 Aug 18;12(36):40339–46. doi: 10.1021/acsami.0c11474 (PMC10905426; doi:10.1021/acsami.0c11474)
Supplement: Supplementary file 1 — am0c11474_si_001.pdf [file am0c11474_si_001.pdf]

Supporting Information
Transient Evolution of Built-in Field at Junctions of GaAs Photoelectrodes

Xihan Chen[1], Ryan T. Pekarek[1], Jing Gu[2], Andriy Zakutayev[1], Katherine Hurst[1], Nathan Neale[1], Ye Yang[3]* and Matthew C. Beard[1]*

1. Materials and Chemical Science and Technology Directorate, National Renewable Energy Laboratory, Golden, Colorado 80401, United States

2. Department of Chemistry and Biochemistry, San Diego State University, San Diego, California 92182, United States

3. State Key Laboratory of Physical Chemistry of Solid Surfaces, College of Chemistry and Chemical Engineering, Xiamen University, Xiamen, Fujian 361005, China

Corresponding Author

Ye Yang* ye.yang@xmu.edu.cn Matthew C. Beard* Matt.Beard@nrel.gov

Materials and experimental methods

GaAs

The GaAs samples studied in this work were purchased from MTI corporation with (100) termination and 0.625 mm thick (Si doped for n-type, Zn-doped for p-type). (Richmond, CA). The complex refractive index at different photon energies was taken from published literature.(Fig. S1)[1] The bandgap of GaAs is close to 1.43 eV and the doping density is ~ 9.1×10^{17} cm^{-3} for n-type and ~ 3×10^{18} cm^{-3} for the p-type sample.

Contact Layers Depositions

Various contacting layers including NiO, TiO_2 or Pt were deposited on the n-GaAs surfaces *via* ALD and sputtering. NiO thin films were deposited by sputtering under similar conditions as described in the literature.[2] In short, NiO was deposited using reactive radio-frequency (RF) sputtering from 50mm-diameter Ni targets at 60W power at 300 °C, in pressure 5×10^{-3} Torr set by flowing 20 sccm of Ar and 6 sccm of O_2, in chamber with base pressure of $<1\times10^{-6}$ Torr. Witness NiO films on glass with 15-40 nm thickness had (111) crystallographic orientation as indicated by XRD (Rigaku D-Max 2200 diffractometer) (Fig. S2), 4-12 S/cm electrical conductivity, and >3 eV optical absorption onset, according to analysis performed using public CombIgor software package[3] (accessible at https://www.combigor.com/). The resulting data for this sample is publicly available in HTEM database at https://htem.nrel.gov/#/samples/6767. The TiO_2 ALD coatings were grown at

300°C, by alternating exposures of titanium isopropoxide (55°C) and water (23°C) in a custom-made viscous flow reactor. This system operates in a continuous flow of high purity N_2 at approximately 0.5 Torr. The pulse length of each precursor was 3s, and 2s respectively, with a purge of 25s in between each pulse. The growth rate of TiO_2 on a Si witness sample under the same conditions had a growth rate of ~.45A/cycle, as measured by ellipsometry (J. A. Woollam M-2000DI). The data were fit using a Cauchey model assuming TiO_2/SiO_2 bilayer structure on Si. The film deposited on the n-GaAs samples consisting of 600 cycles, which equates to approximately 27 nm. The resulting TiO_2 is amorphous. Pt was deposited by ALD by following procedures in previous published literature.[4] Briefly, nanoparticle Pt by alternating exposures of trimethyl(methylcyclopentadienyl)platinum(IV) and a hydrogen plasma. The ALD Pt deposition was performed in a custom-built, plasma-enhanced ALD reactor. The deposition used 2 s doses of trimethyl (methylcyclopentadienyl)platinum(IV) (MeCpPtMe3), alternating with 15 s of 100-W hydrogen plasma exposure. The sample stage temperature was 120 C, and 150 cycles of alternating Pt and H_2 plasma were performed to target a nominal continuous-film thickness equivalent to 2–3 nm.

Molecular Functionalization

The molecular functionalized surface was constructed with 4-(trifluoromethyl)phenyl by a previous established the procedure.[5] Each wafer was cleaned by sonication in a series of solvents (electronic grade acetone, J.T. Baker; isopropanol, Fischer; and deionized water; 10 min each) and etched for 60 s in concentrated H_2SO_4 (J.T. Baker, reagent grade). After etching, the wafer was immediately transferred to a Schlenk flask and brought under a N_2 atmosphere. Dry HCl (2 M in Et_2O (diethyl ether), Aldrich) was added to the flask by canula transfer to submerge the wafer for 50 min. Afterwards, the wafer was rinsed three times with dry Et_2O without air exposure. A solution to bind the molecule to the surface was prepared separately by adding 0.9 equiv. of n-butyl lithium (2.5 M in hexanes, Sigma) to a −78 °C 0.125 M solution of 4-bromobenzotrifluoride in dry THF (Tetrahydrofuran, Sigma). The solution (used immediately upon preparation) was added the flask via canula and allowed to warm to room temperature over 1 h. The solution was then removed and rinsed with THF without air exposure and stored in an Ar glovebox for analysis. For the p-GaAs sample, the as received sample was etched the same way as the functionalized sample and then left in air. A natural oxide terminated surfaces is then formed.

Transient Photoreflectance

A detailed description of the TPR technique can be found elsewhere.[6] Briefly, the TPR measurements were performed by a pump–probe spectrometer (Helios, ultrafast

systems). A Ti:Sapphire amplifier (Libra, Coherent) is used to generate 800 nm light at 1KHz repetition rate. The fundamental pulse is split into two parts. One part is sent to an optical parametric amplifier (TOPAS, Lightconversion) for the various pump wavelength generation. The pump is chopped at a frequency of 500 Hz and attenuated by neutral density filter wheels. The other part of the fundamental pulse is focused into a sapphire crystal to generate a NIR continuum (750–1400 nm) that is used as the probe. The probe pulses are delayed in time with respect to the pump pulses using a motorized translation stage mounted with a retroreflecting mirror. The pump and probe are spatially overlapped on the surface of the sample. The incident angle for pump is around 0° and probe is around 45°. The reflected probe pulses are directed to the multichannel complementary metal–oxide–semiconductor sensor. The size of the focused spot at the sample position for the probe and pump beams is around 200 μm and 600 μm, respectively. Due to reflection geometry, the effective probing depth was around 20 nm ($d_{probe} \cong \lambda/4\pi n$).[6]

Solution to the diffusion equation and modeling of kinetic traces.

1. At t = 0, the initial carrier distribution follows

$$N(x, t = 0) = N_0 e^{\frac{-4\pi k}{\lambda}x} = N_0 e^{-\alpha x} \tag{S1}$$

where α is the absorption coefficient ($\alpha = 4\pi k/\lambda$) for different pump wavelengths and N_0 is the incident photon density or instant carrier density.

2. We divide the carrier distribution into two parts as discussed in the text. Part 1 contains those carriers that reside within the space charge region or the depletion region (w), this portion of carriers are separated by interfacial electric field on a much faster timescale than carrier diffusion. Upon carrier separation they reduce the built-in surface field and add to the photo-modulated reflectance signal. For our simulation we assume these carriers contribute to the signal at delay time of t=0. The number of carriers within the depletion region is given by

$$N(x < w, t = 0) = \int_0^w dx\, N_0 e^{-\alpha x} = \frac{1}{\alpha} N_0 (1 - e^{-\alpha w}) \\ = (1 - e^{-\alpha w}) N_{total} \tag{S2}$$

Where $N_{total} = N_0/\alpha$ is the total carrier density that is absorbed by the sample. To normalize, we can take $\frac{1}{\alpha} N_0 = N_{total} = 1$. For part 2 we calculate the carrier flux that passes through the depletion region boundary (w) as a function of the pump-probe delay time. Carriers that pass through add to the signal as they pass through. The number of carries at the edge of the depletion region at delay time of zero is given by,

$$N(x = w, t = 0) = N_0 e^{-\alpha w} = \alpha e^{-\alpha w} N_{total} \tag{S3}$$

3. For carriers outside the depletion region, they will follow the diffusion equation (equation 1 in the main text $\frac{\partial N}{\partial t} = D\frac{\partial^2 N}{\partial x^2} + \frac{N}{\tau}$). Since the GaAs wafer is much thicker than the light penetration depth, the solution to diffusion equation can be solved analytically:[7]

$$N(x' = 0, t) = N(x = w, t) \times \left(\frac{1}{2}\left(1 + \frac{\alpha D + S_v}{\alpha D - S_v}\right)\omega\left(\alpha\sqrt{Dt}\right) - \frac{S_v}{\alpha D - S_v}\omega\left(S_v\sqrt{\frac{t}{D}}\right)\right) \tag{S4}$$

$$N(x = w, t) = \alpha e^{-\alpha w} N_{total}\left[\frac{\alpha D}{\alpha D - S_v}\omega\left(\alpha\sqrt{Dt}\right) - \frac{S_v}{\alpha D - S_v}\omega\left(S_v\sqrt{\frac{t}{D}}\right)\right]$$

Where here we take $x' = x - w = 0$ when x=w. S_v is the minority carrier thermal velocity defined in the main text, D is the diffusion constant, $\omega\left(\alpha\sqrt{Dt}\right)$ and $\omega\left(S_v\sqrt{\frac{t}{D}}\right)$ are the scaled complementary error function that has the form $\omega(x) = e^{-x^2}erfc(x)$ and is approximated with the following equation[8]

$$\omega(x) = e^{-x^2}erfc(x) \approx \frac{a}{(a-1)\sqrt{\pi x^2} + \sqrt{\pi x^2 + a^2}} \tag{S5}$$

Where a = 2.7889 as suggested in reference S8.

4. The flux of carriers that diffuse from the field free region into the space charge region is given by equation 2 of the main text which can be written out as:

$$J = D\frac{\partial N}{\partial x}|_{x=W_0} = S_v N(x > w, t) = S_v \alpha e^{-\alpha w} N_{total}\left[\frac{\alpha D}{\alpha D - S_v}\omega\left(\alpha\sqrt{Dt}\right) - \frac{S_v}{\alpha D - S_v}\omega\left(S_v\sqrt{\frac{t}{D}}\right)\right] \tag{S6}$$

The total carrier flux that moves into depletion region and adds to the field modulation and thus the measured signal is described by equation 3 of the main text. Therefore, we integrate equation S6 from zero to time t.

$$N(t) = \int_0^t J dt = \int_0^t dt\, S_v \alpha e^{-\alpha w} N_{total} \left[\frac{\alpha D}{\alpha D - S_v} \omega\left(\alpha \sqrt{Dt}\right) - \frac{S_v}{\alpha D - S_v} \omega\left(S_v \sqrt{\frac{t}{D}} \right) \right] \quad \text{(S7)}$$

This integration is solved numerical for each delay time t to map out the simulated dynamical traces.

5. Finally, the total number of carriers inside the depletion regions as a function of time will be the sum of two parts

$$\begin{aligned} N(t) &= N(x < w) + N(t) \\ &= (1 - e^{-\alpha w}) N_{total} \\ &\quad + N_{total} S_v \alpha e^{-\alpha w} \int_0^t dt \left[\frac{\alpha D}{\alpha D - S_v} \omega\left(\alpha \sqrt{Dt}\right) - \frac{S_v}{\alpha D - S_v} \omega\left(S_v \sqrt{\frac{t}{D}} \right) \right] \end{aligned} \quad \text{(S8)}$$

Equation is used to simulate transient kinetics observed in the measurements.

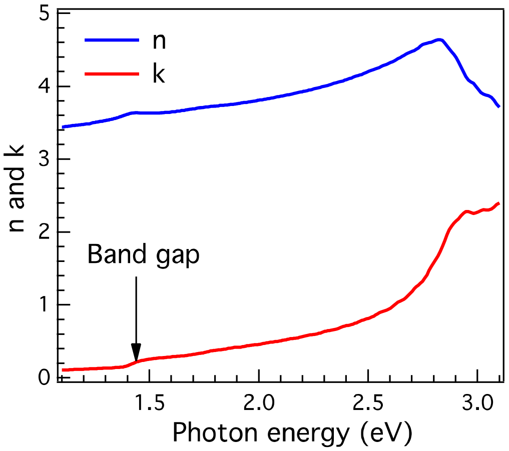

Figure S1. Refractive index (n and k) of GaAs

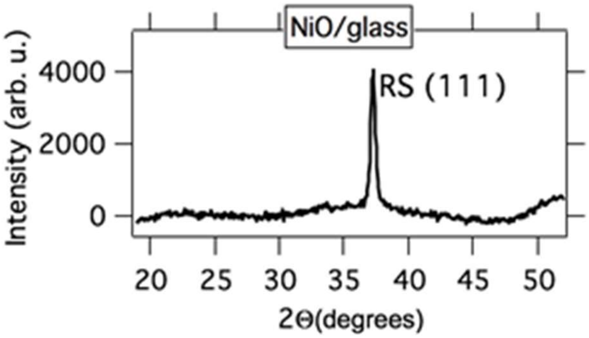

Figure S2. XRD of sputtered NiO film on glass, the XRD pattern shows a 111 phase for sputtered NiO

Carrier density at 2.48 eV pump

Based on the n and k value in figure S1, we can calculate the penetration depth of 2.48 eV pump using $d = \frac{\lambda}{4\pi k}$. And the penetration depth is determined to be 50 nm. The diameter of the pump is ~ 600 μm. Therefore, the pump volume is close to $V = \pi(\frac{D}{2})^2 d = 1.4 \times 10^{-8}\ cm^{-3}$. For a typical experiment, the incident pump fluence is close to 0.5 nJ/pulse. Based on Fresnel equation ($R = |\frac{n_1 - n_2}{n_1 + n_2}|^2$), the absorbed pump is 62.4% based on the complex refractive index n. Therefore, the carrier density is $\rho = \frac{0.5\ nJ \times 62.4\%}{2.48 eV \times 1.4 \times 10^{-8}\ cm^{-3}} = 5.6 \times 10^{-16}\ cm^{-3}$. Other calculated carrier density please see the table below.

Fluence (nJ/pulse)	Carrier Density (cm^{-3})
0.5	5.61×10^{16}
1.2	1.35×10^{17}
2.0	2.25×10^{17}
4.0	4.50×10^{17}
5.3	5.95×10^{17}
8.2	9.21×10^{17}

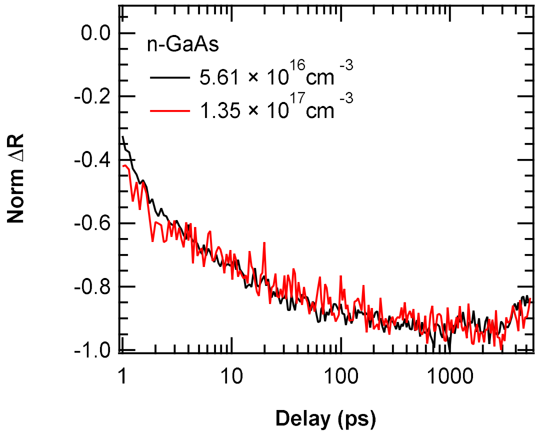

Figure S3. Normalized transient kinetics excited at 2.48 eV for n-GaAs in air with two different carrier density. The TPR kinetics stays the same for low carrier density.

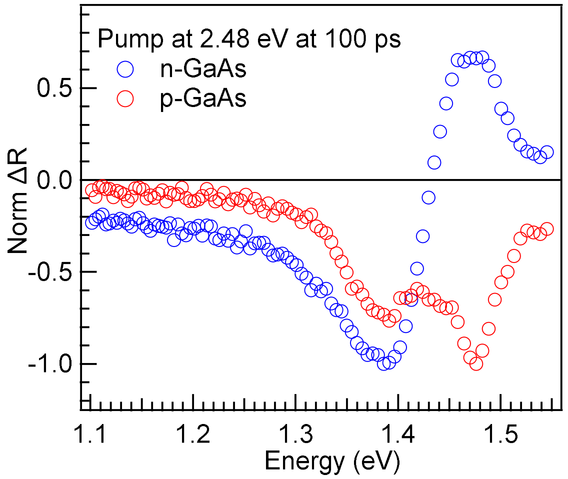

Figure S4. Normalized transient spectra excited at 2.48 eV for n-GaAs and p-GaAs in air at 100 ps delay.

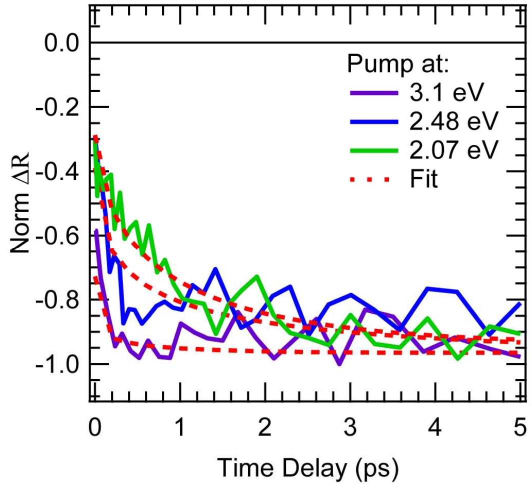

Figure S5. Normalized transient kinetics with fits (rise part) excited at 3.1, 2.48 and 2.07 eV for p-GaAs in air. The data is fitted with the same modelling method described above. For p-type GaAs, the minority carrier is electrons which has much larger diffusion constant ($\sim 150\ cm^2\ s^{-1}$, 20 time larger than n-type), thermal velocity ($\sim 1.8\ \times\ 10^8 cm\ s^{-1}$, 10 times larger) and smaller depletion region width (~25 nm, $\sqrt{2}$ times smaller) than n-type. As seen in Figure 5a, p-type material has a decay component after 5 ps. To account for that in our modelling, we fit the decay part with a single exponential function and the resulting simulation curve in Figure S5 is the product of charge separation kinetics times decay dynamics. We understand this model is not perfect, it is used here try to describe the dynamics in p-GaAs.

(1) Ozaki, S.; Adachi, S. *J. Appl. Phys.* **1995**, *78*, 3380.
(2) Sun, K.; Saadi, F. H.; Lichterman, M. F.; Hale, W. G.; Wang, H.-P.; Zhou, X.; Plymale, N. T.; Omelchenko, S. T.; He, J.-H.; Papadantonakis, K. M.; Brunschwig, B. S.; Lewis, N. S. *Proc. Natl. Acad. Sci.* **2015**, *112*, 3612.

(3) Talley, K. R.; Bauers, S. R.; Melamed, C. L.; Papac, M. C.; Heinselman, K. N.; Khan, I.; Roberts, D. M.; Jacobson, V.; Mis, A.; Brennecka, G. L.; Perkins, J. D.; Zakutayev, A. *ACS Comb. Sci.* **2019**, *21*, 537.

(4) Gu, J.; Yan, Y.; Young, J. L.; Steirer, K. X.; Neale, N. R.; Turner, J. A. *Nat. Mater.* **2016**, *15*, 456.

(5) Garner, L. E.; Steirer, K. X.; Young, J. L.; Anderson, N. C.; Miller, E. M.; Tinkham, J. S.; Deutsch, T. G.; Sellinger, A.; Turner, J. A.; Neale, N. R. *ChemSusChem* **2017**, *10*, 767.

(6) Yang, Y.; Gu, J.; Young, J. L.; Miller, E. M.; Turner, J. A.; Neale, N. R.; Beard, M. C. *Science* **2015**, *350*, 1061.

(7) Beard, M. C.; Turner, G. M.; Schmuttenmaer, C. A. *Phys. Rev. B* **2000**, *62*, 15764.

(8) Ren, C.; MacKenzie, A. R. *Atmos. Sci. Lett.* **2007**, *8*, 70.
